# A Pathway-Based Kernel Boosting Method for Sample Classification Using Genomic Data

**DOI:** 10.3390/genes10090670

**Published:** 2019-08-31

**Authors:** Li Zeng, Zhaolong Yu, Hongyu Zhao

**Affiliations:** 1 Department of Biostatistics, Yale University, New Haven, CT 06511, USA; 2 Interdepartmental Program in Computational Biology and Bioinformatics, Yale University, New Haven, CT 06511, USA

**Keywords:** classification, gene set enrichment analysis, boosting, kernel method

## Abstract

The analysis of cancer genomic data has long suffered “the curse of dimensionality.” Sample sizes for most cancer genomic studies are a few hundreds at most while there are tens of thousands of genomic features studied. Various methods have been proposed to leverage prior biological knowledge, such as pathways, to more effectively analyze cancer genomic data. Most of the methods focus on testing marginal significance of the associations between pathways and clinical phenotypes. They can identify informative pathways but do not involve predictive modeling. In this article, we propose a Pathway-based Kernel Boosting (PKB) method for integrating gene pathway information for sample classification, where we use kernel functions calculated from each pathway as base learners and learn the weights through iterative optimization of the classification loss function. We apply PKB and several competing methods to three cancer studies with pathological and clinical information, including tumor grade, stage, tumor sites and metastasis status. Our results show that PKB outperforms other methods and identifies pathways relevant to the outcome variables.

## 1. Introduction

High-throughput genomic technologies have enabled cancer researchers to study the associations between genes and clinical phenotypes of interest. A large number of cancer genomic data sets have been collected with both genomic and clinical information from the patients. The analyses of these data have yielded valuable insights on cancer mechanisms, subtypes, prognosis and treatment response.

Although many methods have been developed to identify genes informative of clinical phenotypes and build prediction models from these data, it is often difficult to interpret the results with single-gene focused approaches, as one gene is often involved in multiple biological processes and the results are not robust when the signals from individual genes are weak. As a result, pathway-based methods have gained much popularity (e.g., Subramanian et al. [1]). A pathway can be considered as a set of genes that are involved in the same biological process or molecular function. It has been shown that gene-gene interactions may have stronger effects on phenotypes when the genes belong to the same pathway or regulatory network [2]. There are many pathway databases available, such as the Kyoto Encyclopedia of Genes and Genomes [3] (KEGG), the Pathway Interaction Database [4] and Biocarta [5]. By utilizing pathway information, researchers may aggregate weak signals from the same pathway to identify relevant pathways with better power and interpretability. Many pathway-based methods, such as GSEA [1], LSKM [6] and SKAT [7], focus on testing the significance of pathways. These methods consider each pathway separately and evaluate statistical significance for its relevance to the phenotype. In other words, these methods study each pathway separately without considering the effects of other pathways.

Given that many pathways likely contribute to the onset and progression of a disease [8,9,10]. It is of interest to study the contribution of a specific pathway to phenotypes conditional on the effects of other pathways. This is usually achieved by regression models. Wei and Li [11] and Luan and Li [12] proposed two similar models, Nonparametric Pathway-based Regression (NPR) and Group Additive Regression (GAR). Both models employ a boosting framework, construct base learners from individual pathways and perform prediction through additive models. Due to the additivity at the pathway level, these models only considered interactions among genes within the same pathway but not across pathways. Since our proposed method is motivated by the above two models, more details of these models will be described in Section 2. In genomics data analysis, multiple kernel methods [13,14] are also commonly used when predictors have group structures. In these methods, one kernel is assigned to each group of predictors and a meta-kernel is computed as a weighted sum of the individual kernels. The kernel weights are estimated through optimization and can be considered as a measure of pathway importance. Multiple kernel methods have been used to integrate multi-pathway information or multi-omics data sets and have achieved state-of-the-art performance in predictions of various outcomes [15,16,17].

In this paper, we propose a Pathway-based Kernel Boosting (PKB) method for sample classification. In our boosting framework, we use the second order approximation of the loss function instead of the first order approximation used in the usual gradient descent boosting method, which allows for deeper descent at each step. We introduce two types of regularizations ($L_{1}$ and $L_{2}$) for selection of base learners in each iteration and propose algorithms for solving the regularized problems. In Section 3.1, we conduct simulation studies to evaluate the performance of PKB, along with four other competing methods. In Section 3.2, we apply PKB to three cancer genomics data sets, where we use gene expression data to predict several patient phenotypes, including tumor grade, stage, tumor site and metastasis status.

## 2. Materials and Methods

Suppose our observed data are collected from *N* subjects. For subject *i*, we use a *p* dimensional vector $x_{i}=\left(x_{i1},x_{i2},\ldots ,x_{ip}\right)$ to denote the normalized gene expression profile and $y_{i}\in \left\{1,-1\right\}$ to denote its class label. Similarly, the gene expression levels of a given pathway *m* with $p_{m}$ genes can be represented by $x_{i}^{\left(m\right)}=\left(x_{i1}^{\left(m\right)},x_{i2}^{\left(m\right)},\ldots ,x_{ip_{m}}^{\left(m\right)}\right)$, which is a sub-vector of $x_{i}$.

The log loss function is commonly used in binary classifications with the following form:

$$l\left(y,F\left(x\right)\right)=\log\left(1+e^{-yF(x)}\right),$$

and is minimized by

$$F^{*}\left(x\right)=\log\frac{p(y=1|x)}{p(y=-1|x)},$$

which is exactly the log odds function. Thus the sign of an estimated F(x) can be used to classify sample x as 1 or −1. Since genes within the same pathway likely have much stronger interactions than genes in different pathways, in our pathway-based model setting, we assume additive effects across pathways and focus on capturing gene interactions within pathways:

$$F\left(x\right)=\sum_{m=1}^{M}H_{m}\left(x^{\left(m\right)}\right),$$

where each $H_{m}$ is a nonlinear function that only depends on the expression levels of genes in the *m*th pathway and summarizes its contribution to the log odds function. Due to the additive nature of this model, it only captures gene interactions within each pathway but not across pathways.

Two existing methods, NPR [11] and GAR [12], employed the Gradient Descent Boosting (GDB) framework [18] to estimate the functional form of F(x) nonparametrically. GDB can be considered as a functional gradient descent algorithm to minimize the empirical loss function, where in each descent iteration, an increment function that best aligns with the negative gradient of the loss function (evaluated at each sample point) is selected from a space of base learners and then added to the target function F(x). NPR and GAR extended GDB to be pathway-based by applying the descent step to each pathway separately and selecting the base learner from the pathway that provides the best fit to the negative gradient.

NPR and GAR differ in how they construct base learners from each pathway: NPR uses regression trees and GAR uses linear models. Due to the linearity assumption of GAR, it lacks the ability to capture complex interactions among genes in the same pathway. Using regression tree as base learners enables NPR to model interactions, however, there is no regularization in the gradient descent step, which can lead to selection bias that prefers larger pathways.

Motivated by NPR and GAR, we propose the PKB model, where we employ kernel functions as base learners, optimize loss function with second order approximation [19] which gives Newton-like descent speed and also incorporates regularization in selection of pathways in each boosting iteration.

### 2.1. PKB Model

Kernel methods have been applied to a variety of statistical problems, including classification [20], regression [21], dimension reduction [22] and others. Results from theories of Reproducing Kernel Hilbert Space [23] have shown that kernel functions can capture complex interactions among features. For pathway *m*, we construct a kernel-based function space as the space for base learners

$$G_{m}=\left\{g\left(x\right)=\sum_{i=1}^{N}K_{m}\left(x_{i}^{\left(m\right)},x_{}^{\left(m\right)}\right)\beta_{i}+c:\beta_{1},\beta_{2},\ldots ,\beta_{N},c\in R\right\},$$

where $K_{m}\left(\cdot ,\cdot \right)$ is a kernel function that defines similarity between two samples only using genes in the *m*th pathway. The overall base learner space is the union of the spaces constructed from each pathway alone: $G=\cup_{m=1}^{M}G_{m}$.

Estimation of the target function F(x) is obtained through iterative minimization of the empirical loss function evaluated at the observed data. The empirical loss is defined as

$$L\left(y,F\right)=\frac{1}{N}\sum_{i=1}^{N}l\left(y_{i},F\left(x_{i}\right)\right),$$

where $F=(F\left(x_{1}\right),F\left(x_{2}\right),\ldots ,F\left(x_{N}\right))$. In the rest of this article, we will use the bold font of a function to represent the vector of the function evaluated at the observed $x_{i}$’s. Assume that at iteration *t*, the estimated target function is $F_{t}\left(x\right)$. In the next iteration, we aim to find the best increment function f∈G and add it to $F_{t}\left(x\right)$. Expanding the empirical loss at $F_{t}$ to the second order, we can get the following approximation

(1) $$L_{\mathrm{approx}}\left(y,F_{t}+f\right)=L\left(y,F_{t}\right)+\frac{1}{N}\sum_{i=1}^{N}\left[h_{t,i}f\left(x_{i}\right)+\frac{1}{2}q_{t,i}f{\left(x_{i}\right)}^{2}\right],$$

where

$$\begin{array}{ccc} h_{t,i} & = & \frac{\partial L(y,F_{t})}{\partial F_{t}\left(x_{i}\right)}=-\frac{y_{i}}{1+e^{y_{i}F_{t}\left(x_{i}\right)}},\\ q_{t,i} & = & \frac{\partial^{2}L\left(y,F_{t}\right)}{\partial F_{t}{\left(x_{i}\right)}^{2}}=\frac{e^{y_{i}F_{t}\left(x_{i}\right)}}{{\left(1+e^{y_{i}F_{t}\left(x_{i}\right)}\right)}^{2}} \end{array}$$

are the first order and second order derivatives with respect to each $F_{t}\left(x_{i}\right)$, respectively. We propose a regularized loss function that incorporates both the approximated loss and a penalty on the complexity of *f*:

(2)LR(f)=Lapprox(y,Ft+f)+Ω(f)(3)=1N∑i=1Nqi,t2(hi,tqi,t+f(xi))2+Ω(f)+C(y,Ft),

where Ω(·) is the penalty function. Since f∈G is a linear combination of kernel functions calculated from a specific pathway, the norm of the combination coefficients can be used to define Ω(·). We consider both $L_{1}$ and $L_{2}$ norm penalties and solutions regarding each penalty option are presented in Section 2.1.1 and Section 2.1.2, respectively. $C(y,F_{t})$ is a constant term with respect to *f*. Therefore, we only use the first two terms of Equation (3) as the working loss function in our algorithms. We will also drop $C(y,F_{t})$ in the expression of $L_{R}\left(f\right)$ in the following sections for brevity. Such a penalized boosting step has been employed in several methods (e.g., Johnson and Zhang [24]). Intuitively, the regularized loss function would prefer simple solutions that also fit the observed data well, which usually leads to better generalization capability to unseen data.

We then optimize the regularized loss for the best increment direction

$$\hat{f}=\arg\min_{f\in G}L_{R}\left(f\right).$$

Given the direction, we find the deepest descent step length by minimizing over the original loss function

$$\hat{d}=\arg\min_{d\in R^{+}}L\left(y,F_{t}+\hat{d}\hat{f}\right),$$

and update the target function to $F_{t+1}\left(x\right)=F_{t}\left(x\right)+\nu \hat{d}\hat{f}$, where ν is a learning rate parameter. The above fitting procedure is repeated until a certain pre-specified number of iterations is reached. The complete procedure of the PKB algorithm is shown in Table 1.

#### 2.1.1. $L_{1}$ Penalized Boosting

The core step of PKB is the optimization of the regularized loss function (see step 3 of Table 1). Note that G is the union of the pathway-based learner spaces, thus

$$\begin{array}{ccc} \hat{f} & = & \arg\min_{G}L_{R}\left(f\right)\\ & = & \arg\min_{{\hat{f}}_{m}}\;\left\{L_{R}\left({\hat{f}}_{m}\right):{\hat{f}}_{m}=\arg\min_{f\in G_{m}}L_{R}\left(f\right),m=1,2,\ldots ,M\right\}. \end{array}$$

To solve for $\hat{f}$, it is sufficient to obtain the optimal ${\hat{f}}_{m}$ in each pathway-based subspace. Due to the way we construct the subspaces, in a given pathway *m*, *f* takes a parametric form as a linear combination of the corresponding kernel functions. This helps us further reduce the optimization problem to

(4)minf∈GmLR(f)=minβ,c1N∑i=1Nqi,t2(hi,tqi,t+Km,iTβ+c)2+Ω(f)(5)=minβ,c1N(ηt+Kmβ+1Nc)TWt(ηt+Kmβ+1Nc)+Ω(f),

where

$$\begin{array}{ccc} \eta_{t} & = & {\left(\frac{h_{1,t}}{q_{1,t}},\frac{h_{2,t}}{q_{2,t}},\ldots ,\frac{h_{N,t}}{q_{N,t}}\right)}^{T},\\ W_{t} & = & \mathrm{diag}(\frac{q_{1,t}}{2},\frac{q_{2,t}}{2},\ldots ,\frac{q_{N,t}}{2}),\\ K_{m} & = & {\left[K_{m}\left(x_{i}^{\left(m\right)},x_{j}^{\left(m\right)}\right)\right]}_{i,j=1,2,\ldots ,N}. \end{array}$$

$K_{m,i}$ is the *i*th column of kernel matrix $K_{m}$ and $1_{N}$ is an *N* by 1 vector of 1’s. We use the $L_{1}$ norm $\Omega \left(f\right)={\lambda ∥\beta ∥}_{1}$, as the penalty term, where λ is a tuning parameter adjusting the amount of penalty we impose on model complexity. We also prove that after certain transformations, the optimization can be converted to a LASSO problem without intercept

(6) $$\min_{\beta }\frac{1}{N}∥\tilde{\eta }+{\tilde{K}}_{m}{\beta ∥}_{2}^{2}+\lambda {∥\beta ∥}_{1},$$

where

$$\begin{array}{ccc} \tilde{\eta } & = & W_{t}^{\frac{1}{2}}\left[I_{N}-\frac{1_{N}1_{N}^{T}W_{t}}{tr(W_{t})}\right]\eta_{t}\\ {\tilde{K}}_{m} & = & W_{t}^{\frac{1}{2}}\left[I_{N}-\frac{1_{N}1_{N}^{T}W_{t}}{tr(W_{t})}\right]K_{m}. \end{array}$$

Therefore, β can be efficiently estimated using existing LASSO solvers. The proof of the equivalence between the two problems is provided in Section 1 of the Supplementary Materials.

#### 2.1.2. $L_{2}$ Penalized Boosting

In the $L_{2}$ penalized boosting, we replace Ω(f) in the objective function of (5) with ${\lambda ∥\beta ∥}_{2}^{2}$. Following the same transformation as that in Section 2.1.1, the objective can also be converted to a standard Ridge Regression (see Section 1 of Supplementary Materials)

(7) $$\min_{\beta }\frac{1}{N}∥\tilde{\eta }+{\tilde{K}}_{m}{\beta ∥}_{2}^{2}+\lambda {∥\beta ∥}_{2}^{2},$$

which allows closed form solution

$$\hat{\beta }=-{\left({\tilde{K}}_{m}^{T}{\tilde{K}}_{m}+N\lambda I_{N}\right)}^{-1}{\tilde{K}}_{m}^{T}\tilde{\eta }.$$

Both the $L_{1}$ and $L_{2}$ boosting algorithms require the specification of the penalty parameter λ, which controls step length (the norm of fitted β) in each iteration and additionally controls solution sparsity in the $L_{1}$ case. Feasible choices of λ might be different for different scenarios, depending on the input data and also the choice of the kernel. Either too small or too large λ values would lead to big leaps or slow descent speed. Under the $L_{1}$ penalty, poor choices of λ can even result in all-zero β, which makes no change to the target function. Therefore, we also incorporate an optional automated procedure to choose the value of λ in PKB. Computational details of the procedure are provided in Section 2 of the Supplementary Materials. We recommend the use of the automated procedure to calculate a feasible λ and try a range of values around it (e.g., the calculated value multiplies 1/25,1/5,1,5,25) for improved performance.

Lastly, the final target function at iteration *T* can be written as

$$F_{T}\left(x\right)=\sum_{m=1}^{M}\sum_{i=1}^{N}K_{m}\left(x_{i}^{\left(m\right)},x_{}^{\left(m\right)}\right)\beta_{i}^{\left(m\right)}+C,$$

where $\beta_{}^{\left(m\right)}=\left(\beta_{1}^{\left(m\right)},\beta_{2}^{\left(m\right)},\ldots ,\beta_{N}^{\left(m\right)}\right)$ are the combination coefficients of kernel functions from pathway *m*. We use $∥\beta_{}^{\left(m\right)}{∥}_{2}$ as a measure of importance (or weight) in the target function. It is obvious that only the pathways that are selected at least once in the boosting procedure will have non-zero weights. Because $F_{T}\left(x\right)$ is an estimation of the log odds function, $\mathrm{sign}[F_{T}\left(x\right)]$ is used as the classification rule to assign x to 1 or −1.

## 3. Results

### 3.1. Simulation Studies

We use simulation studies to assess the performance of PKB. We consider the following three underlying true models:

- Model 1:
  $$F\left(x\right)=2x_{1}^{\left(1\right)}+3x_{2}^{\left(1\right)}+\exp\left(0.8x_{1}^{\left(2\right)}+0.8x_{2}^{\left(2\right)}\right)+4x_{1}^{\left(3\right)}x_{2}^{\left(3\right)}$$
- Model 2:
  $$F\left(x\right)=4\sin\left(x_{1}^{\left(1\right)}+x_{2}^{\left(1\right)}\right)+3\left|x_{1}^{\left(2\right)}-x_{2}^{\left(2\right)}\right|+2{x_{1}^{\left(3\right)}}^{2}-2{x_{2}^{\left(3\right)}}^{2}$$
- Model 3:
  $$F\left(x\right)=2\sum_{m=1}^{10}{∥x^{\left(m\right)}∥}_{2}$$
  where F(x) is the true log odds function and $x_{i}^{\left(m\right)}$ represents the expression level of the *i*th gene in the *m*th pathway. We include different functional forms of pathway effects in F(x), including linear, exponential, polynomial and others. In models 1 and 2, only two genes in each of the first three pathways are informative to sample classes; in model 3, only genes in the first ten pathways are informative. We generated a total of six datasets, two for each model, with different numbers of irrelevant pathways (M = 50 and 150) corresponding to different noise levels. We set the size of pathways to 5 and sample size to 900 in all simulations. Gene expression data ($x_{i}$’s) were generated following standard normal distribution. We then calculated the log odds $F(x_{i})$ for each sample and use the median-centered $F(x_{i})$ values to generate corresponding binary outcomes $y_{i}\in \{$−1, 1} (We usethe median-centered $F(x_{i})$ values to generate outcome, so that the proportions of 1’s and −1’s are approximately 50%.).

We divided the generated datasets into three folds and each time used two folds as training data and the other fold as testing data. The number of maximum iterations *T* is important to PKB, as using a large *T* will likely induce overfitting on training data and poor prediction on testing data. Therefore, we performed nested cross validation within the training data to select *T*. We further divided the training data into three folds and each time trained the PKB model using two folds while monitoring the loss function on the other fold at every iteration. Eventually, we identified the iteration number $T^{*}$ with the minimum averaged loss on testing data and applied PKB to the whole training dataset up to $T^{*}$ iterations.

**Figure 1:**
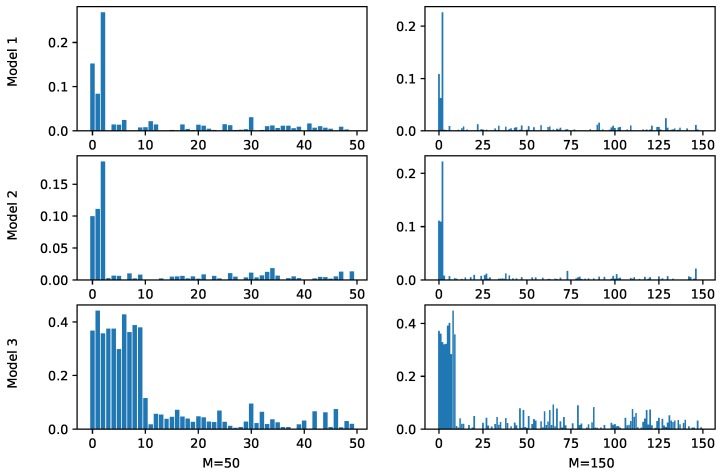
Estimated pathway weights by PKB in simulation studies. The X-axis represents pathways and the Y-axis represents estimated weights. Based on the simulation settings, the first three pathways are relevant in Models 1 and 2 and the first ten pathways are relevant in Model 3. *M* represents the number of simulated pathways.

We first evaluated the ability of PKB to correctly identify relevant pathways. For each simulation scenario, we calculated the average optimal weights across different cross validation runs and the results are shown in Figure 1, where the X-axis represents different pathways and the length of bars above them represents corresponding weights in the prediction functions. Note that for the underlying Model 1 and Model 2, only the first three pathways were relevant to the outcome, and in Model 3, the first ten pathways were relevant. In all the cases, PKB successfully assigned the largest weights to relevant pathways. Since PKB is an iterative approach, at some iterations, certain pathways irrelevant to the outcome may be selected by chance and added to the prediction function. This explains the non-zero weights of the irrelevant pathways and their values are clearly smaller than those of relevant pathways.

We also applied several commonly used methods to the simulated datasets and compared their prediction accuracy with PKB. These methods included both non-pathway-based methods: Random Forest [25] and SVM [20] and pathway-based methods: NPR [11] and EasyMKL [14]. Model parameters we used for the above methods are listed in Section 3 of the Supplementary Materials. We used the same three-fold split of the data, as we used when applying PKB, to perform cross-validations for each competing method. The average prediction performance of the methods is summarized in Table 2. It can be seen that the pathway-based methods generally performed better than the non-pathway-based methods in all simulated scenarios. Among the pathway-based methods, the one that utilized kernels (EasyMKL) had comparable performances with the tree-based NPR method in Models 1 and 2 but had clearly superior performance in Model 3. This was likely due to the functional form of the log odds function F(x) of Model 3. Note that genes in relevant pathways were involved in F(x) in terms of their $L_{2}$ norms, which is hard to approximate by regression tree functions but can be well captured using kernel methods. In all scenarios, the best performance was achieved by one of the PKB methods. In four out of six scenarios, the PKB-$L_{2}$ method produced the smallest prediction errors, while in the other two scenarios, PKB-$L_{1}$ was slightly better. Although PKB-$L_{1}$ and PKB-$L_{2}$ had similar performances, PKB-$L_{1}$ was usually computationally faster, because in the optimization step of each iteration, the $L_{1}$ algorithm only looked for sparse solution of β’s, which can be done more efficiently than PKB-$L_{2}$, which involves matrix inverse.

### 3.2. Real Data Applications

We applied PKB to gene expression profiles to predict clinical features in three cancer studies, including breast cancer, melanoma and glioma. The clinical variables we considered included tumor grade, tumor site and metastasis status, which were all of great importance to cancer.

We used three commonly used pathway databases: KEGG, Biocarta and Gene Ontology (GO) Biological Process pathways. These databases provide lists of pathways with emphasis on different biological aspects, including molecular interactions and involvement in biological processes. The number of pathways from these databases ranges from 200 to 700. There is considerable overlap between pathways. To eliminate redundant information and control the overlap between pathways, we applied a preprocessing step to the databases with details provided in the Supplementary Materials Section 4.2.

Similar to the simulation studies, we compared the performances from different methods based on three fold cross validations following the same procedure as elaborated in Section 3.1. Most of the methods we considered have tuning parameters. We searched through different parameter configurations and reported the best result from cross-validation for each method. More details of the data sets and the implementations can be found in the Supplementary Materials Section 4. Table 3 shows the classification error rates from all methods. The numbers in bold are the optimal error rates for each column separately. In four out of five classifications, PKB was the best method (usually with the $L_{1}$ and $L_{2}$ methods being the top two). In the other case (melanoma, stage), NPR yielded the best results, with the PKB methods still ranking second and third.

We provide more detailed introductions to the data sets and clinical variables and interpretations of results by PKB in the following. For brevity of the article, we focus on presenting results for three outcomes, one from each data set and leave the other two in the Supplementary Materials (Section 4.4).

#### 3.2.1. Breast Cancer

Metabric is a breast cancer study that involved more than 2000 patients with primary breast tumors [26]. The data set provides copy number aberration, gene expression, mutation and long-term clinical follow-up information. We are interested in the clinical variable of tumor grade, which measures the abnormality of the tumor cells compared to normal cells under a microscope. It takes a value of 1, 2, or 3. Higher Grade indicates more abnormality and higher risk of rapid tumor proliferation. Since grade 1 contained the fewest samples, we pooled it together with Grade 2 as one class and treated Grade 3 as the other class.

We then applied PKB to samples in subtype Lum B, where the sample sizes for the two classes were most balanced (259 Grade 3 patients; 211 Grade 1,2 patients). For input gene expression data, we used the normalized mRNA expression (microarray) data for 24,368 genes provided in the data set. The model using GO Biological Process pathways and radial basis function (rbf) kernel yielded the best performance (error rate 27.4%). To obtain the pathways most relevant to tumor grade, we calculated the average pathway weights from the cross validation and sorted them from highest to lowest. Top fifteen pathways with the highest weights are presented in the first columns of Table 4.

Among all pathways, the cell aggregation and sequestering of metal ion pathways are the top two pathways in terms of the estimated pathway weights. Previous research has shown that cell aggregation contributes to the inhibition of cell death and anoikis-resistance, thereby promoting tumor cell proliferation. Genes in the cell aggregation pathway include TGFB2, MAPK14, FGF4 and FGF6, which play important roles in the regulation of cell differentiation and fate [27]. Moreover, the majority of genes in the sequestering of metal ion pathway encode calcium-binding proteins, which regulates calcium level and different cell signaling pathways relevant to tumorigenesis and progression [28]. Among these genes, S100A8 and S100A9 have been identified as novel diagnostic markers of human cancer [29]. The results suggest that PKB has identified pathways that are likely relevant to breast cancer grade.

#### 3.2.2. Lower Grade Glioma

Glioma is a type of cancer developed in the glial cells in brain. As glioma tumor grows, it compresses normal brain tissue and can lead to disabling or fatal results. We applied our method to a lower Grade glioma data set from TCGA, where only grades 2 and 3 samples were collected (Grade 4 glioma, also known as glioblastoma, is studied in a separate TCGA study.) [30]. After removal of missing values, the numbers of patients in the cohort with grades 2 and 3 tumors were 248 and 265, respectively. We used Grade as the outcome variable to be classified and applied PKB with different parameter configurations. After cross validation, PKB using the third order polynomial (poly3) kernel and the GO Biological Process pathways yielded an error rate of 28.3%, which was the smallest among all methods. The top fifteen pathways selected in the model are listed in the second column of Table 4.

The estimated pathway weights indicate that the cell adhesion pathway and the neuropeptide signaling pathway have the strongest association with glioma grade. Genes in the cell adhesion pathways generally govern the activities of cell adhesion molecules. Turning off the expression of cell-cell adhesion molecules is one of the hallmarks of tumor cells, by which tumor cells can inhibit antigrowth signals and promote proliferation. Previous studies have shown that deletion of carcinoembryonic antigen-related cell adhesion molecule 1 (CEACAM1) gene can contribute to cancer progression [31]. Cell Adhesion Molecule 1 (CADM1), CADM2, CADM3 and CADM4, serve as tumor suppressors and can inhibit cancer cell proliferation and induce apoptosis. Neuropeptide signaling pathway has also been implicated in tumor growth and progression. Neuropeptide Y is highly relevant to tumor cell proliferation and survival. Two NPY receptors, Y2R and Y5R, are also members of the neuropeptide signaling pathway. They are considered as important stimulatory mediators in tumor cell proliferation [32].

#### 3.2.3. Melanoma

The next application of PKB is to a TCGA cutaneous melanoma dataset [33]. Melanoma is most often discovered after it has metastasized and the skin melanoma site is never found. Therefore, the majority of the samples are metastatic. In this data set, there are 369 metastatic samples and 103 primary samples. It is of great interest to study the genomic differences between the two types, thus we applied PKB to this data using metastatic/primary as the outcome variable. Using the Biocarta pathways and rbf kernel produced the smallest classification error rate (8.1%) among all methods. Fifteen pathways that PKB found most relevant to the outcome are presented in the third column of Table 4.

Two complement pathways, lectin induced complement pathway and classical complement pathway, came out from the PKB model as the most significant pathways. Proteins in complement system participate in a variety of biological processes of metastasis, such as epithelial-mesenchymal transition (EMT). EMT is an important process in the initiation stage of metastasis, through which cells in primary tumor lose cell-cell adhesion and gain invasive properties. Complement activation by tumor cells can recruit stromal cells to the tumor and induce EMT. Furthermore, complement proteins can mediate the degradation of extracellular matrix, thereby promoting tumor metastasis [34].

## 4. Discussion

In this paper, we have introduced the PKB model as a method to perform classification analysis of gene expression data, as well as identify pathways relevant to the clinical outcomes of interest. PKB usually yields sparse models in terms of the number of pathways, which enhances interpretability of the results. Moreover, the pathway weights as defined in Section 2 can be used as a measure of pathway importance and provides guidance for further experimental verifications.

Two types of regularizations are introduced in the optimization step of PKB, in order to select simple model with good fitting. Computation efficiency of the two methods depends on the regularization strengh: when regularization is strong, the $L_{1}$ method enjoys a computational advantage due to the sparsity of its solution; when regularization is weak, it requires more iterations to converge and yields worse run time than the $L_{2}$. In simulations and real data applications, both methods yielded comparable prediction accuracy. It is worth mentioning that the second-order approximation of the log loss function is also necessary for efficiency of PKB. The approximation yields an expression that is quadratic in terms of coefficients β, which allows the problem to be converted to LASSO or Ridge Regression after regularizations are added. If the original loss function was used, solving β would be more time consuming. In the applications, we only considered gene expression data as model input. However, our method can be easily generalized to use other continuous inputs, such as gene methylation measurements. By incorporating other properly designed kernel functions, it is also possible to handle discrete inputs (for example, the weighted IBS kernel for SNP data [7]).

There are several limitations of the current PKB approach. First of all, when constructing base learners from pathways, we use fixed bandwidth parameters (inverse of the number of genes in each pathway) in the kernel functions. Ideally, we would like the model to auto-determine the parameters. However, the number of such parameters is equal to the number of pathways, which is often too large to tune efficiently. Therefore, it remains a challenging task for future research. Second, we currently only use pathway as a criterion to group genes and within each pathway, all genes are treated equally. It is conceivable that the genes interact with each other through an underlying interaction network and intuitively, genes in the hub should get more weights compared to genes on the periphery. With the network information available, it is possible to build more sensible kernel functions as base learners [17]. Third, the pathway databases only cover a subset of the input genes. Both KEGG and Biocarta only include a few thousands of genes, while the number of input genes is usually beyond 15,000. Large number of genes, with the potential to provide additional prediction power, remain unused in the model. In our applications, we tried pooling together all unused genes and consider them as a new pathway but it did not significantly improve the results. Although genes annotated with pathways are supposed to be most informative, it is still worth looking for smarter ways of handling unannotated genes.

## Figures and Tables

**Table 1:** An overview of the Pathway-based Kernel Boosting (PKB) algorithm。

1. Initialize target function as an optimal constant:
$F_{0}\left(x\right)=\arg\min_{r\in R}\frac{1}{n}\sum_{i=1}^{N}l\left(y_{i},r\right)$
For t from 0 to T-1 (maximum number of iterations) do:
2. calculate the first and second derivatives:
$h_{t,i}=-\frac{y_{i}}{1+e^{y_{i}F_{t}\left(x_{i}\right)}},q_{t,i}=\frac{e^{y_{i}F_{t}\left(x_{i}\right)}}{{\left(1+e^{y_{i}F_{t}\left(x_{i}\right)}\right)}^{2}}$
3. optimize the regularized loss function in the base learner space:
$\hat{f}=\arg\min_{f\in G}L_{R}\left(f\right)$
4. find the step length with the steepest descent:
$\hat{d}=\arg\min_{d\in R^{+}}L\left(y,F_{t}+d\hat{f}\right)$
5. update the target function:
$F_{t+1}\left(x\right)=F_{t}\left(x\right)+\nu \hat{d}\hat{f}\left(x\right)$
End For
return $F_{T}\left(x\right)$

**Table 2:** Classification error rate from PKB and competing methods in simulation studies. The numbers below each model represent the number of pathways simulated in the data sets.

Method	Model 1			Model 2			Model 3	
	50	150		50	150		50	150
PKB-$L_{1}$	**0.151**	0.196		**0.198**	0.189		0.179	0.21
PKB-$L_{2}$	0.158	**0.185**		0.201	**0.183**		**0.157**	**0.173**
Random Forest	0.305	0.331		0.290	0.328		0.341	0.400
SVM	0.353	0.431		0.412	0.476		0.431	0.492
NPR	0.271	0.321		0.299	0.317		0.479	0.440
EasyMKL	0.253	0.284		0.268	0.330		0.212	0.300

**Table 3:** Classification error rates on real data. The names in the parenthesis of each data set are the variables used as classification outcome. The best error rates are highlighted with bold font for each column.

Method	Data Sets				
	Metabric (Grade)	Glioma (Grade)	Glioma (Site)	Melanoma (Stage)	Melanoma (Met)
PKB-$L_{1}$	**0.274**	**0.283**	0.168	0.304	**0.081**
PKB-$L_{2}$	0.304	**0.283**	**0.154**	0.307	0.083
Random Forest	0.306	0.302	0.306	0.320	0.136
SVM	0.285	0.292	0.185	0.314	0.083
NPR	0.306	0.298	0.197	**0.282**	0.110
EasyMKL	0.297	0.302	0.291	0.314	0.100

**Table 4:** Top fifteen pathways with the largest weights fitted by PKB. In each column, pathways are sorted in descending order from top to bottom. Pathways in the first two columns are from GO Biological Process pathways and the third column from Biocarta.

	Metabric (Grade)	Glioma (Grade)	Melanoma (Met)
1	Cell aggregation	Homophilic cell adhesion via plasma membrane adhesion molecules	Lectin induced complement pathway
2	Sequestering of metal ion	Neuropeptide signaling pathway	Classical complement pathway
3	Glutathione derivative metabolic process	Multicellular organismal macromolecule metabolic process	Phospholipase c delta in phospholipid associated cell signaling
4	Antigen processing and presentation of exogenous peptide antigen via mhc class i	Peripheral nervous system neuron differentiation	Fc epsilon receptor i signaling in mast cells
5	Sterol biosynthetic process	Positive regulation of hair cycle	Inhibition of matrix metalloproteinases
6	Pyrimidine containing compound salvage	Peptide hormone processing	Regulation of map kinase pathways through dual specificity phosphatases
7	Protein dephosphorylation	Hyaluronan metabolic process	Estrogen responsive protein efp controls cell cycle and breast tumors growth
8	Homophilic cell adhesion via plasma membrane adhesion molecules	Positive regulation of synapse maturation	Chaperones modulate interferon signaling pathway
9	Cyclooxygenase pathway	Stabilization of membrane potential	Il-10 anti-inflammatory signaling pathway
10	Establishment of protein localization to endoplasmic reticulum	Lymphocyte chemotaxis	Reversal of insulin resistance by leptin
11	Negative regulation of dephosphorylation	Insulin secretion	Bone remodeling
12	Xenophagy	Positive regulation of osteoblast proliferation	Cycling of ran in nucleocytoplasmic transport
13	Attachment of spindle microtubules to kinetochore	Negative regulation of dephosphorylation	Alternative complement pathway
14	Fatty acyl coa metabolic process	Trophoblast giant cell differentiation	Cell cycle: g${}^{2}$/m checkpoint
15	Apical junction assembly	Synaptonemal complex organization	Hop pathway in cardiac development

## Supplementary Materials

Supplementary materials and reproduction code are available online at https://github.com/zengliX/PKB. Reproduction-related input data sets are available upon request from the corresponding author (hongyu.zhao@yale.edu).

## Abbreviations

The following abbreviations are used in this manuscript:

KEGG	Kyoto Encyclopedia of Genes and Genomes
TCGA	The Cancer Genome Atlas

## References

[1] Subramanian A., Tamayo P., Mootha V.K., Mukherjee S., Ebert B.L., Gillette M.A., Paulovich A., Pomeroy S.L., Golub T.R., Lander E.S. (2005). Gene set enrichment analysis: A knowledge-based approach for interpreting genome-wide expression profiles. Proc. Natl. Acad. Sci. USA.

[2] Carlson C.S., Eberle M.A., Kruglyak L., Nickerson D.A. (2004). Mapping complex disease loci in whole-genome association studies. Nature.

[3] Kanehisa M., Goto S. (2000). KEGG: Kyoto encyclopedia of genes and genomes. Nucleic Acids Res..

[4] Schaefer C.F., Anthony K., Krupa S., Buchoff J., Day M., Hannay T., Buetow K.H. (2008). PID: The pathway interaction database. Nucleic Acids Res..

[5] Nishimura D. (2001). BioCarta. Biotech Softw. Internet Rep. Comput. Softw. J. Sci..

[6] Liu D., Lin X., Ghosh D. (2007). Semiparametric Regression of Multidimensional Genetic Pathway Data: Least-Squares Kernel Machines and Linear Mixed Models. Biometrics.

[7] Wu M.C., Lee S., Cai T., Li Y., Boehnke M., Lin X. (2011). Rare-variant association testing for sequencing data with the sequence kernel association test. Am. J. Hum. Genet..

[8] Shou J., Massarweh S., Osborne C.K., Wakeling A.E., Ali S., Weiss H., Schiff R. (2004). Mechanisms of tamoxifen resistance: Increased estrogen receptor-HER2/neu cross-talk in ER/HER2–positive breast cancer. J. Natl. Cancer Inst..

[9] Shtivelman E., Hensing T., Simon G.R., Dennis P.A., Otterson G.A., Bueno R., Salgia R. (2014). Molecular pathways and therapeutic targets in lung cancer. Oncotarget.

[10] Berk M. (2009). Neuroprogression: Pathways to progressive brain changes in bipolar disorder. Int. J. Neuropsychopharmacol..

[11] Wei Z., Li H. (2007). Nonparametric pathway-based regression models for analysis of genomic data. Biostatistics.

[12] Luan Y., Li H. (2007). Group additive regression models for genomic data analysis. Biostatistics.

[13] Gönen M., Margolin A.A. (2014). Drug susceptibility prediction against a panel of drugs using kernelized Bayesian multitask learning. Bioinformatics.

[14] Aiolli F., Donini M. (2015). EasyMKL: A scalable multiple kernel learning algorithm. Neurocomputing.

[15] Costello J.C., Heiser L.M., Georgii E., Gönen M., Menden M.P., Wang N.J., Bansal M., Hintsanen P., Khan S.A., Mpindi J.P. (2014). A community effort to assess and improve drug sensitivity prediction algorithms. Nat. Biotechnol..

[16] Friedrichs S., Manitz J., Burger P., Amos C.I., Risch A., Chang-Claude J., Wichmann H.E., Kneib T., Bickeböller H., Hofner B. (2017). Pathway-based kernel boosting for the analysis of genome-wide association studies. Comput. Math. Methods Med..

[17] Manica M., Cadow J., Mathis R., Martínez M.R. (2019). PIMKL: Pathway-Induced Multiple Kernel Learning. NPJ Syst. Biol. Appl..

[18] Friedman J.H. (2001). Greedy function approximation: A gradient boosting machine. Ann. Stat..

[19] Friedman J., Hastie T., Tibshirani R. (2000). Additive logistic regression: A statistical view of boosting (with discussion and a rejoinder by the authors). Ann. Stat..

[20] Guyon I., Weston J., Barnhill S., Vapnik V. (2002). Gene selection for cancer classification using support vector machines. Mach. Learn..

[21] Drucker H., Burges C.J., Kaufman L., Smola A.J., Vapnik V. (1997). Support Vector Regression Machines. Advances in Neural Information Processing Systems.

[22] Fukumizu K., Bach F.R., Jordan M.I. (2009). Kernel dimension reduction in regression. Ann. Stat..

[23] Friedman J., Hastie T., Tibshirani R. (2001). The Elements of Statistical Learning.

[24] Johnson R., Zhang T. (2014). Learning nonlinear functions using regularized greedy forest. IEEE Trans. Pattern Anal. Mach. Intell..

[25] Breiman L. (2001). Random forests. Mach. Learn..

[26] Pereira B., Chin S.F., Rueda O.M., Vollan H.K.M., Provenzano E., Bardwell H.A., Pugh M., Jones L., Russell R., Sammut S.J. (2016). The somatic mutation profiles of 2,433 breast cancers refines their genomic and transcriptomic landscapes. Nat. Commun..

[27] Zhang X., Xu L.h., Yu Q. (2010). Cell aggregation induces phosphorylation of PECAM-1 and Pyk2 and promotes tumor cell anchorage-independent growth. Mol. Cancer.

[28] Monteith G.R., McAndrew D., Faddy H.M., Roberts-Thomson S.J. (2007). Calcium and cancer: Targeting Ca^2+^ transport. Nat. Rev. Cancer.

[29] Hermani A., Hess J., De Servi B., Medunjanin S., Grobholz R., Trojan L., Angel P., Mayer D. (2005). Calcium-binding proteins S100A8 and S100A9 as novel diagnostic markers in human prostate cancer. Clin. Cancer Res..

[30] TCGA (2015). Comprehensive, integrative genomic analysis of diffuse lower-grade gliomas. N. Engl. J. Med..

[31] Leung N., Turbide C., Olson M., Marcus V., Jothy S., Beauchemin N. (2006). Deletion of the carcinoembryonic antigen-related cell adhesion molecule 1 (Ceacam1) gene contributes to colon tumor progression in a murine model of carcinogenesis. Oncogene.

[32] Tilan J., Kitlinska J. (2016). Neuropeptide Y (NPY) in tumor growth and progression: Lessons learned from pediatric oncology. Neuropeptides.

[33] Akbani R., Akdemir K.C., Aksoy B.A., Albert M., Ally A., Amin S.B., Arachchi H., Arora A., Auman J.T., TCGA (2015). Genomic classification of cutaneous melanoma. Cell.

[34] Pio R., Corrales L., Lambris J.D. (2014). The Role of Complement in Tumor Growth. Tumor Microenvironment and Cellular Stress.

